# First Usutu Virus Infection in an Asymptomatic Blood Donor in Greece

**DOI:** 10.3390/tropicalmed11050138

**Published:** 2026-05-18

**Authors:** Anna Papa, Katerina Tsioka, Styliani Pappa, Danai Pervanidou, Constantina Politis, Kostas Stamoulis, Vassiliki Bakaloudi

**Affiliations:** 1 Department of Microbiology, Medical School, Aristotle University of Thessaloniki, 54124 Thessaloniki, Greece; aik.tsioka@gmail.com (K.T.); s_pappa@hotmail.com (S.P.); 2 National Public Health Organisation, 15123 Athens, Greece; d.pervanidou@eody.gov.gr (D.P.); cpolitis11@yahoo.gr (C.P.); 3 Hellenic National Blood Transfusion Center, 13672 Athens, Greece; kostas.stamoulis@gmail.com; 4 Blood Center, AHEPA University General Hospital, 54636 Thessaloniki, Greece; bakaloudi@gmail.com

**Keywords:** Usutu virus, blood donor, whole genome sequence, phylogenetic analysis, Greece

## Abstract

Usutu virus (USUV) is a mosquito-borne flavivirus, widely distributed in Central Europe, where it causes avian outbreaks with large-scale mortality. Although most human infections are asymptomatic or mild, the reports of USUV neurologic infections are increasing, especially among immunocompromised patients. Cross-reactivity in serological and molecular assays is often seen between USUV and the genetically and antigenically related West Nile virus (WNV). Here, we report the first USUV infection in Greece in an asymptomatic blood donor who tested positive in the automated nucleic acid test during screening for WNV, which is endemic in the country. Following the blood donation surveillance plan, a serum sample taken two weeks post-donation was tested for WNV IgM and IgG antibodies. The borderline index of the IgM antibodies, combined with negative result for IgG antibodies, raised the suspicion of molecular cross-reactivity with USUV. Although the USUV-specific PCR in donor’s plasma was negative, its result was positive following amplification of the virus in cell culture, as USUV RNA was detected in the culture supernatant confirming the USUV infection. Whole genome sequence was taken using an Ion Torrent next-generation sequencing platform. Phylogenetic analysis showed that the Greek strain clusters within the USUV Europe 2A genetic lineage. The detection of USUV human infection in Greece prompts for surveillance studies to elucidate its epidemiology and ecology in the country.

## 1. Introduction

Usutu virus (USUV, *Orthoflavivirus usutuense*) is circulating in nature between *Culex* spp. mosquitoes as vectors and birds as amplifying hosts, while humans and horses are dead-end hosts with short-lasting and low-level viraemia [1]. The virus was first isolated in 1959 from *Culex neavei* mosquitoes collected near the Usutu River in South Africa [2,3], while the first two human infections were identified in the Central African Republic and Burkina Faso in 1981 and 2004, respectively [4]. In Europe, USUV was initially detected in 2001–2002 in dead birds in Austria [5], while a retrospective study showed that the virus was already present in archived tissue samples from dead birds collected in 1996 in the Tuscany region of Italy [6]. Several avian outbreaks (particularly in blackbirds) with mass mortality were observed in Europe [6,7,8]. Human USUV infections are usually asymptomatic or present as mild febrile illness often accompanied by rash; however, an increasing number of neuroinvasive cases are being reported, mainly in immunocompromised patients, while in rare cases, the disease has a fatal outcome [9,10,11,12,13]. Several USUV infections have been reported in blood donors who had been tested positive in the automated nucleic acid test (NAT) systems during routine West Nile virus (WNV) screening [14,15,16,17,18,19]. This is due to cross-reactivity between these two genetically related viruses as they are both belonging to the Japanese encephalitis serocomplex of the *Orthoflavivirus* genus in the *Flaviviridae* family [20].

USUV has a positive-sense single-stranded RNA genome with a length of approximately 11,000 nucleotides, which encodes a polyprotein of around 3400 amino acids. Currently, the USUV sequences cluster into eight lineages, which are named on the basis of their geographic origin of isolation: Europe (EU)1 to EU5, and Africa (AF)1 to AF3; all lineages, except AF1, have been detected in Europe, suggesting several virus introductions in the continent [8].

Greece is one of the European countries with the highest annual incidence of human WNV infections. However, there is not any report of human USUV infection. The first serological evidence of USUV circulation in Greece was in November 2010 when a domestic pigeon sampled in Central Macedonia Region in the frame of a WNV bird surveillance survey was found positive in WNV ELISA, but negative in WNV neutralization test, while it was positive for USUV neutralizing antibodies [21]. Studies in *Culex pipiens* mosquitoes showed that the circulation of USUV is low. In a mosquito surveillance study conducted during 2020–2022 in three Regions of Greece (Attica, Central Macedonia and Thessaly), USUV was detected in four of 1500 pools (0.27%) of *C. pipiens* mosquitoes; the positive mosquitoes had been trapped in late August 2020 and in September 2022, all in the Central Macedonia Region in northern Greece [22]. In a recent study, USUV was detected in one of 1316 (0.07%) pools of *C. pipiens* mosquitoes collected in 2024 in various administrative Regions in Greece; the mosquitoes of the positive pool had been trapped in September [23]. This specific pool was one of the 737 pools (0.13%) of mosquitoes collected in Central Macedonia Region. The partial USUV sequence was clustering into the EU2 lineage [23]. Here, we report the first USUV infection in a Greek asymptomatic blood donor and we present the phylogenetic relationships based on whole genome sequence of the strain.

## 2. Materials and Methods

In late September 2024, a female asymptomatic donor with no comorbidities at her late 20s was found repeatedly WNV positive by NAT. The donor lived in Central Macedonia in northern Greece, with no travel outside that region during the incubation period. The NAT was performed at the Blood Center of AHEPA University General Hospital in Thessaloniki using the Cobas 8800 System (Roche Diagnostics International AG, Rotkreuz, Switzerland). Following the WNV-positive results, the blood unit was discarded.

According to the applied algorithm for blood donors screening, a NAT reactive result initiates a series for further testing, e.g., retest the initial plasma sample by NAT, and test a follow-up serum sample taken 14 days after donation by NAT and by serology at the National Reference Laboratory for Arboviruses and hemorrhagic fever viruses in Aristotle University of Thessaloniki for the presence of WNV-specific IgM and IgG antibodies. An additional serum sample taken 28 days after donation was also tested as the serological result of the previous sample was not indicative of WNV infection (Table 1). Up to that time, the donor remained asymptomatic.

### 2.1. Serological Testing for West Nile Virus

Two serial serum samples taken from the blood donor 14 and 28 weeks after donation were tested. For the detection of WNV IgM and IgG antibodies the WNV IgM capture DxSelect and WNV IgG DxSelect commercial ELISA kits (Focus Diagnostics Inc., Cypress, CA, USA) were used. According to the manufacturers, an index >1.1 for IgM and >1.5 for IgG is defined as positive result. The IgG avidity was measured in the IgG positive samples using the same ELISA IgG kit and 6 M urea; avidity value <50% is defined as low avidity, suggestive of a recent WNV infection [24].

### 2.2. Virus Isolation

Vero E6 cell monolayers were inoculated with 350 ul donors’ plasma in a 25 cm^2^ flask containing 10 mL culture medium consisting of minimum essential medium with 10% fetal bovine serum, L-glutamine 200 mM, penicillin and streptomycin 5000 U/mL, amphotericin B 250 ug/mL and sodium bicarbonate solution 7.5%. All culture reagents were purchased from Gibco (ThermoFisher Scientific, Waltham, MA, USA). The flask was incubated at 37 °C with 5% CO_2_, and the presence of cytopathic effects (CPEs), such as cell rounding and detachment, was monitored daily. After three serial passages of the virus in new flasks, the virus stocks were stored at −80 °C. All the procedures for USUV isolation were performed in the biosafety level 3 laboratory by staff trained to work within this containment level.

### 2.3. Molecular Testing

Viral RNA was extracted from 140 ul blood donor’s plasma and from culture supernatant using the QIAamp Viral RNA kit (Qiagen, Hilden, Germany). The testing for WNV RNA was performed using a commercial real-time RT-PCR kit (Sacace Biotechnologies, Como, Italy). For the detection of USUV RNA, an in-house USUV-specific real-time RT-PCR was applied using the primer set USU-F and USU-R2 and the probe USU-S [25].

### 2.4. Next-Generation Sequencing

De novo next-generation sequencing (NGS) was performed on the viral RNA extracted from the culture supernatant. First-strand cDNA synthesis was performed from 10 ul of RNA using SuperScript III Reverse Transcriptase (Invitrogen, ThermoFisher Scientific, Waltham, MA, USA) and oligo (dT) primers; second-strand synthesis was carried out using Klenow fragment DNA polymerase (New England Biolabs, Ipswich, MA, USA).

The libraries were prepared using the Ion 510 & Ion 520 & Ion 530 for Ion Chef kit for 400 base-reads and quantified using the Ion Library TaqMan Quantitation kit. Then, they were normalized to a final concentration of 30 pM, and the library (single end) was loaded in an Ion 520 semiconductor sequencing chip on an Ion Torrent S5 platform following the manufacturer’s instructions. All reagents were obtained from ThermoFisher Scientific. Raw reads were processed for quality control through the Torrent Suite software version 5.18.1, and the obtained sequences were aligned using the sequence of USUV strain Vienna 2001 (GenBank Accession number NC_006551) as reference. Assembly and annotation of the USUV whole genome sequence were performed using Geneious Prime, version 2021.2.1.

### 2.5. Phylogenetic Analysis

The USUV whole genome sequence was aligned with respective sequences obtained from the GenBank Database using CLUSTAL W, and a phylogenetic tree was generated using the maximum likelihood method and Tamura-Nei model of nucleotide substitutions in MEGA version 12 software [26].

## 3. Results

### 3.1. WNV NAT Screening

The results from the NAT screening of the donor’s samples in the Blood Center of AHEPA University General Hospital in Thessaloniki are seen in Table 1.

### 3.2. Serological and Molecular Testing

The results of the serological and molecular testing in the Reference Laboratory are seen in Table 2. Specifically, the index of the WNV IgM antibodies in the serum sample taken 14 days after donation was at the borderline, while WNV IgG antibodies were not detected, and the WNV-specific PCR was negative. Therefore, a second serum sample was taken 14 days later (28 days post donation). Although WNV IgG antibodies were detected with avidity 20.8% (less than 50%, suggestive of acute infection), WNV IgM antibodies were not detected. Since the serology results were inconclusive, USUV was suspected, and the stored original plasma sample of the donor was tested by an USUV-specific real-time RT-PCR which resulted negative.

### 3.3. Virus Isolation

CPEs were observed in the flasks on the 5th day post-inoculation. The real-time RT-PCR performed on the RNA extracted from the cell culture supernatant gave a positive result with cycle threshold (Ct) 16.5 (Table 2).

### 3.4. Next-Generation Sequencing

A total of 265,070 reads were taken from NGS. The whole genome sequence of USUV consisted of 10,947 nucleotides with an open reading frame encoding the virus polyprotein consisting of 3434 amino acids.

### 3.5. Phylogenetic Analysis

**Figure 1:**
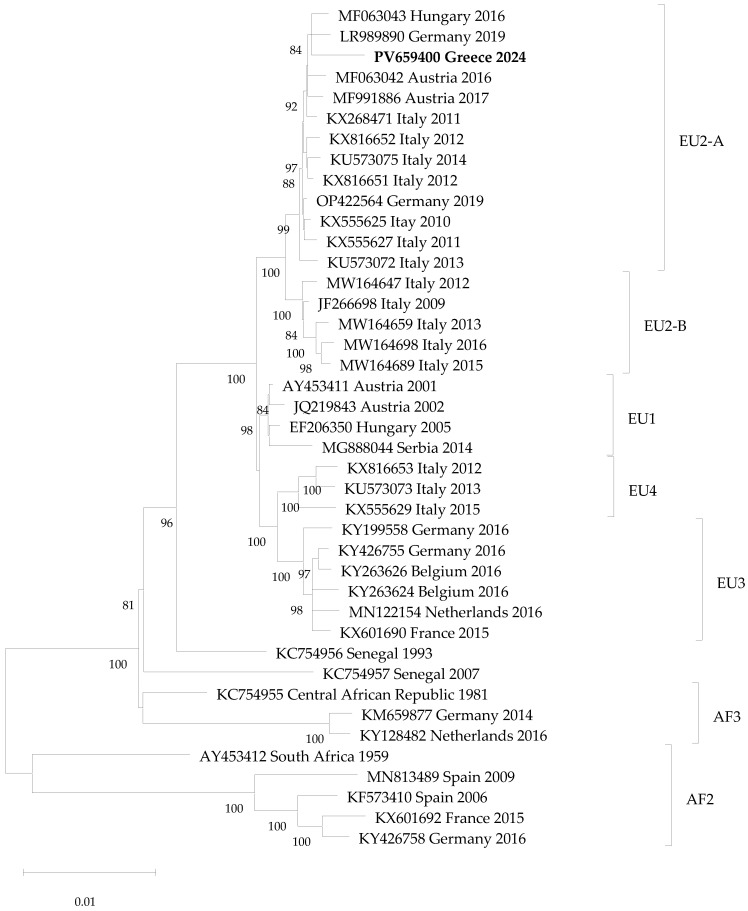
Maximum likelihood phylogenetic tree based on USUV whole genome sequences. The sequence of the present study is shown in bold. The sequences are shown as accession number, country and year of detection.

A maximum likelihood phylogenetic tree based on whole genome sequences of USUV (10,302 nucleotides) is seen in Figure 1. The percentage of replicate trees in which the associated taxa clustered together (1000 replicates) is shown below the branches; only values >75% are shown. The Greek sequence clusters within the USUV EU2 genetic lineage, and specifically in the sub-lineage EU2-A, sharing >99.5% nucleotide identity with European strains of the same sub-lineage.

## 4. Discussion

USUV is an emerging arbovirus in Europe with increasing spatial distribution and number of human infection reports. Most USUV infections are asymptomatic or mild and remain undiagnosed. However, there are several reports of USUV asymptomatic cases which were diagnosed following a false positive result during WNV NAT screening in blood donations due to cross-reactivity between USUV and WNV. Similar cross-reactivity is seen also in serology.

Since 2010 when WNV emerged in Greece, it causes seasonal outbreaks of human infections almost every year [27]. Therefore, the National Public Health Organisation implements annually (during May–November) enhanced surveillance of human WNV infections. An intersectoral Working Group (under the Ministry of Health) designates the “affected” and “high-risk” municipalities (administrative units), where targeted blood safety and hemovigilance measures are implemented. A municipality is defined as “affected” when at least one human case of WNV infection (either with WNND or not) has been recorded during the current season, and as “high risk” when no case has been recorded during the current transmission season, but risk factors are present (e.g., neighboring/adjacent to affected municipalities). The screening programs are applied to donors residing or having visited “affected” and “high-risk” areas. In these areas, the National blood safety authorities have issued guidelines: (i) the blood safety measures include 28-day deferral of donors who spent even one night in an area with ongoing WNV transmission to humans, and WNV ID-NAT blood screening; (ii) the hemovigilance measures include post donation and post transfusion information, as well as retrospective procedures aiming at tracing recipients of blood components from a potentially infectious blood donation; and (iii) the serological follow-up includes test for WNV IgM and IgG antibodies of donors positive for WNV RNA. A retrospective screening of the available blood units taken up to 15 days prior to the municipality’s characterization is also performed in the affected areas, while in high-risk areas the screening starts upon the characterization without retrospective screening.

The donor of the present study lived in an already affected area of Central Macedonia Region, and all measures targeting the affected areas were in place; this is why the donated blood was tested for WNV. The reports of USUV in *C. pipiens* mosquitoes and USUV-specific neutralizing antibodies in a sentinel bird were also from Central Macedonia [21,22,23]. The detection of USUV only in the northern part of the country is probably the result of environmental or other factors rather than a bias in sampling.

The positive WNV NAT result combined with inconclusive results in WNV serology (borderline index of IgM antibodies with no detection of IgG antibodies), prompted for USUV testing. USUV RNA was not detected in the original plasma sample, but it was detected in the culture supernatant taken five days post-inoculation of donor’s plasma in Vero E6 cells, which is explained by the fact that the virus was amplified in the cells at detectable level (Ct 16.5). Four weeks after the donation, WNV IgM antibodies were undetectable in the donor’s serum, but WNV IgG antibodies were detected. USUV serology was not performed; therefore, IgG cross-reactivity between USUV and WNV was not directly proven, but it is assumed based on other studies which have shown that it is often seen between these two flaviviruses even in neutralizing tests [28]. Since the neutralizing antibodies usually target the antigenically similar envelope protein E, they are broadly cross-reactive, highlighting the need for diagnostic tools with increased specificity. A recent study showed that differentiation between recent WNV and USUV infections is possible using quantitative WNV and USUV NS1-based IgM and IgG ELISAs, which should be performed side-by-side to minimize cross-reactivity effects [29].

The Greek USUV strain belongs to EU2-A lineage. It can be seen that the strain isolated in 1993 in Senegal (ArD101291, accession number KC754956) is more closely related to the European USUV strains than to African strains, supporting the hypothesis that USUV was introduced into Europe from Africa, most probably via viremic migratory birds [30]. A recent study estimated that Europe 2 lineage has arisen from an ancestor that existed in Austria following its introduction from Africa between 1970 and the 1980s [31]. The authors mentioned that the fact that many intervening countries in Africa are unsampled, the prediction of spatial movements of USUV between African countries and between Africa and Europe is difficult, and caution is needed in the interpretation of phylogeography results.

While Greece is endemic for WNV, this is the first report of autochthonous USUV human infection in the country. This fact, together with the low prevalence of USUV in mosquitoes, could be attributed to unfavorable ecological conditions not supporting the intense, widespread amplification of USUV as seen in Central European countries [22]. An additional factor may be the interaction and competion between USUV and WNV, since it was shown in cell lines that USUV is outcompeted by WNV in mammalian, avian and mosquito cells [32]. A third explanation is the misdiagnosis of USUV cases due to cross-reactivity with WNV, and the underestimation due to asymptomatic or mild infection. A recent study showed that, in contrast to WNV, USUV cannot infect motor neurons in healthy individuals due to its restriction by the antiviral immune response, which could explain the differences in the clinical impact of these two viruses [33].

Currently, there are no specific regulations for screening blood donations for USUV. However, blood transfusion is often applied to immunosuppressed or severely ill patients with a potential risk for development of nosocomial USUV-associated neurological disease in USUV endemic countries that screen only for WNV [17]. To the best of our knowledge, there is not any report of transfusion-transmitted USUV infection. Further surveillance studies are needed to find out to what extent USUV is implicated in transfusion safety and in public health in general.

## 5. Conclusions

WNV and USUV belong to the same serocomplex of orthoflaviviruses, and cross-reactivity is often seen in the diagnostic assays. Therefore, the differential diagnosis between WNV and USUV infections is challenging, and awareness is needed among clinicians and laboratory professionals not to overlook or misidentify the USUV infections. The detection of autochthonous USUV human infection in Greece prompts for surveillance studies to elucidate its epidemiology and ecology in the country.

## Figures and Tables

**Table 1:** WNV NAT results of the blood donor’s samples.

Sample	Time of Collection	Result/Cycle Threshold
Plasma (blood unit)	Day of donation	Positive/31.99
Plasma (donor)	Day of donation	Positive/30.06
Plasma (donor) *	Day of donation	Positive/31.46
Serum (donor)	15 days post donation	Negative

* Same donor’s plasma sample re-tested.

**Table 2:** Serological and molecular results on samples taken from the blood donor and from culture supernatant taken five days post-inoculation.

Sample	Days Post Donation	WNV IgM	WNV IgG/Avidity	WNV PCR	USUV Real-Time RT-PCR/Cycle Threshold
Stored plasma	0	N.D. ^1^	N.D.	Negative	Negative
Serum	14	0.96 (borderline)	negative	N.D.	N.D.
Serum	28	negative	2.2/20.8	N.D.	N.D.
Culture supernatant		-	-	Negative	Positive/16.5

^1^ N.D.: not done.

## Data Availability

The sequence of the study was submitted to the GenBank DataBase under the accession number PV659400.
